# Bone mineral density changes in osteoporotic and osteopenic patients after COVID-19 infection

**DOI:** 10.1186/s43166-022-00165-7

**Published:** 2022-12-13

**Authors:** Samah Hamdy Elmedany, Omaima Ibrahim Badr, Mohammed Hassan Abu-Zaid, Samar Abd Alhamed Tabra

**Affiliations:** 1 Department of Rheumatology and Rehabilitation, Faculty of Medicine, Tanta University, Tanta, Egypt; 2 Department of Rheumatology and Rehabilitation Medicine, Al Noor Specialist Hospital, Mecca, Saudi Arabia; 3 Department of Chest Medicine, Faculty of Medicine, Mansoura University, Mansoura, Egypt; 4 Department of Pulmonary Medicine, Al Noor Specialist Hospital, Mecca, Saudi Arabia

**Keywords:** Osteoporosis, COVID-19, Bone mineral density, Osteoporosis treatment

## Abstract

**Background:**

Coronavirus disease 2019 (COVID-19) infection is linked to high levels of inflammatory cytokines and prolonged immobilization; furthermore, corticosteroid treatment leads to increased bone loss and resorption. We aimed to study the change in bone mineral density (BMD) after COVID-19 infection in osteoporotic and osteopenic patients. One hundred osteoporotic or osteopenic patients were selected in this single-center retrospective study; the patients were divided into two groups. Group 1 included 56 patients who got COVID-19 infection. Group 2 included 44 patients who did not get COVID-19 infection. BMD was assessed at baseline, after 9 months of COVID infection, and then after 1 year follow-up using dual energy x-ray absorptiometry (DXA) scan.

**Results:**

There was no significant difference between two groups regarding demographic data (*p* > 0.05); there was a significant decrease in BMD of the lumbar region and femur at 9 months as compared to baseline in group1 (*p* < 0.001), while there was a significant increase in the lumbar BMD of osteoporotic patients who did not get COVID infection after 21 months. Concerning activity of COVID infection, there was a significant difference between the three subgroups of COVID patients regarding percentage of change in BMD after 9 months, the severe group having the highest decrease in BMD (*p* < 0.001).

**Conclusions:**

COVID-19 may have deleterious effect on BMD in osteoporotic patients. It is recommended to assess BMD in osteoporotic/osteopenic patients who got COVID infection to detect if there is an increased risk of fracture which may necessitate post-COVID change in the therapeutic intervention plan.

**Supplementary Information:**

The online version contains supplementary material available at 10.1186/s43166-022-00165-7.

## Background

Osteoporosis is the commonest bone disease worldwide, and it is characterized by decreased bone mass with increased fragility fracture risk. Osteoporotic fracture is associated with short-term and long-term morbidity including increased pain, decreased health-related quality of life, and increased mortality [1].

The global coronavirus disease 2019 (COVID-19) pandemic has greatly affected many aspects of medical care including the prevention and care of osteoporosis [2]. The United States Center for Disease Control and Prevention (CDC) recommended prioritizing urgent visits and delaying elective care to decrease the spread of COVID-19 (https://www.cdc.gov/coronavirus/2019-ncov/hcp/facility-planning-operations.html).

COVID-19 infection is linked to high levels of inflammatory cytokines and prolonged immobilization, especially in critically ill patients; furthermore, corticosteroid treatment for treatment of COVID infection and its serious comorbidities increase the risk of bone loss and resorption [3].

Because of the prioritization of urgent services and delaying of elective care, the screening, diagnosis, and management of many chronic medical conditions including osteoporosis have been affected. Many primary care and specialty clinics were temporarily closed, paused, or slowed schedules for screening dual-energy x-ray absorptiometry (DXA) scans. Also, access to osteoporosis treatments can be delayed or missed, especially intravenous or subcutaneous antiresorptive drugs [4].

Many questions regarding the interaction between osteoporosis and COVID-19 and COVID-19 treatment remain unclear. To our knowledge, there is no previous study that investigated the effect of COVID 19 infection on bone mineral density in osteoporotic patients.

The aim of this study is to study the change in bone mineral density (BMD) after COVID-19 infection in osteoporotic and osteopenic patients.

## Methods

### Study design

This is a single-center retrospective study.

### Study setting

Patients were selected from the osteoporosis clinic department of Rheumatology and Rehabilitation Medicine, Al Noor Specialist Hospital, Mecca, Saudi Arabia.

### Participants

One hundred patients who were diagnosed to have osteoporosis or osteopenia (based on *T*-score results, osteoporosis was defined as a *T*-score of BMD ≤  − 2.5, and osteopenia was defined as − 2.5 < *T*-score ≤  − 1) (https://www.osteoporosis.foundation/patients/diagnosis) were selected.

The patients were divided into two groups:

- Group 1: Fifty-six patients with osteoporosis or osteopenia who got COVID-19 infection which was diagnosed and confirmed via polymerase chain reaction.
- Group 2: Forty-four patients with osteoporosis or osteopenia who did not get COVID-19 infection.

Group 1 was divided according to COVID-19 severity scale proposed by the National Institutes of Health into mild, moderate, and severe subgroups defined as follows (https://www.Covid19treatmentguidelines.nih.gov/overview/clinical/spectrum). *Mild*: Individuals who do not have shortness of breath, dyspnea, or abnormal chest imaging. *Moderate*: Individuals who show evidence of lower respiratory disease during clinical assessment or imaging and who have an oxygen saturation (SpO_2_) ≥ 94% on room air at sea level. *Severe*: Individuals who have SpO_2_ < 94% on room air at sea level, a ratio of arterial partial pressure of oxygen to fraction of inspired oxygen (PaO_2_/FiO_2_) < 300 mm Hg, a respiratory rate > 30 breaths/min, or lung infiltrates > 50%.

### Exclusion criteria

We excluded patients with chronic diseases interfering with calcium, phosphorus, and vitamin D metabolism (hyper- or hypoparathyroidism, chronic renal or liver insufficiency, cancer).

### Compliance with ethics guidelines

This study is in agreement with the ethical guidelines of the Declaration of Helsinki, and it follows the ethical standards according to ICH GCP (International Council for Harmonisation, Good Clinical Practice) guidelines, with IRB Number: H-02-K-076–0122-645. Privacy of all patients’ data was granted as there was a code number for every patient file that includes all investigations.

### Assessment

Data were obtained from medical files and electronic records using a distinctive medical record number. Demographic and clinical information of the patients (age, gender, height, weight, smoking, drug history, and associated comorbidities) as well as clinical data for COVID patients (admission, duration of admission, corticosteroid treatment, and its duration and complications of COVID-19) were obtained.

The following laboratory tests were collected from files of all patients at baseline (within 3 months before COVID 19 infection): serum levels of calcium, vitamin D, phosphorus, glycosylated hemoglobin (HbA1c), urea, and creatinine. Serum ferritin was also recorded for the group of patients who got COVID infection.

BMD was assessed at baseline (within 3 months of COVID infection), after 9 months of COVID infection, and then after 1 year of follow-up using the same dual-energy x-ray absorptiometry (DXA) equipment (Lunar DPX densitometer). A trained osteoporosis technician performed all the standardized BMD measurements at the hip (femoral neck and total hip) and lumbar spine L1–L4. The lumbar spine was measured from L1 to L4, and the mean lumbar BMD (L2–L4) was calculated. The left hip was measured. If the left hip could not be measured, the right hip was measured.

### Statistical analysis

Data were fed to the computer and analyzed using the IBM SPSS software package version 20.0. (Armonk, NY: IBM Corp). Categorical data were represented as numbers and percentages. Chi-square test was applied to investigate the association between the categorical variables. For continuous data, they were tested for normality by the Kolmogorov–Smirnov and Shapiro–Wilk test. Quantitative data were expressed as range (minimum and maximum), mean, standard deviation, and median. Student *t*-test was used to compare two groups for normally distributed quantitative variables, while ANOVA with repeated measures was used to compare between more than two periods and post hoc test (Bonferroni adjusted) for pairwise comparisons. On the other hand, Mann–Whitney test was used to compare two groups for not normally distributed quantitative variables. Kruskal–Wallis test was used to compare more than two groups for not normally distributed quantitative variables. The results were considered statistically significant at *p* ≤ 0.05.

## Results

**Fig. 1:**
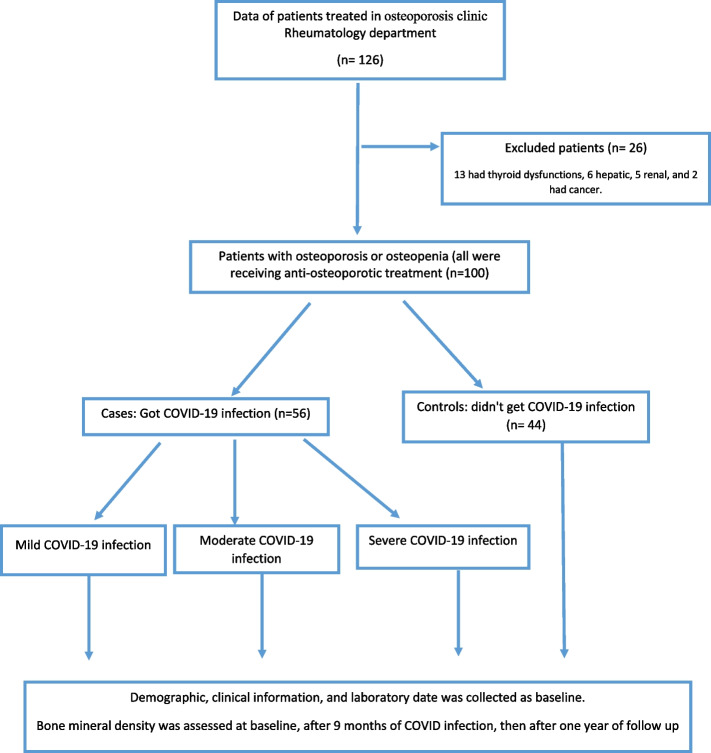
Consort flow diagram of the case control study

A total of one hundred patients diagnosed to have osteoporosis or osteopenia were included. Baseline patients’ characteristics for demographic and patients’ clinical characteristics were shown in Table 1; all patients were receiving osteoporotic treatment. There was no statistically significant difference between the two groups regarding age, sex, and BMI. Figure 1 shows consort flow diagram of the case control study.

**Table 1:** Demographic and clinical characteristics data of the patients (*n* = 100)

	**Total (*n* = 100)**	**COVID (*n* = 56)**	**Non-COVID (*n* = 44)**	**Test of Sig**	***p***
**Age (years)**					
Mean ± SD	62.31 ± 7.88	62.84 ± 9.05	61.64 ± 6.10	*t* = 0.792	0.431
**Sex**					
Male	26 (26.0%)	15 (26.8%)	11 (25.0%)	*χ*^2^ = 0.041	0.840
Female	74 (74.0%)	41 (73.2%)	33 (75.0%)		
**BMI (kg/m**^**2**^**)**					
Mean ± SD	28.48 ± 5.75	28.34 ± 6.03	28.67 ± 5.44	*t* = 0.283	0.778
**Total physical activity**					
Inactive	62 (62.0%)	36 (64.3%)	26 (59.1%)	*χ*^2^ = 0.282	0.595
Moderately active	38 (38.0%)	20 (35.7%)	18 (40.9%)		
Active	0 (0.0%)	0 (0.0%)	0 (0.0%)		
**Smoking**	30 (30.0%)	15 (26.8%)	15 (34.1%)	*χ*^2^ = 0.626	0.429
**Comorbidity**					
No comorbidity	18 (18.0%)	8 (14.3%)	10 (22.7%)	*χ*^2^ = 2.575	0.63
Rheumatologic disease	43 (43.0%)	24 (42.8%)	19 (43.2%)		
Cardiac	32 (32.0%)	17 (30.4%)	15 (34.1%)		
HTN	60 (60.0%)	36 (64.3%)	24 (54.5%)		
DM	50 (50.0%)	32 (57.1%)	18 (40.9%)		
**Osteoporosis vs. osteopenia**					
Osteopenia	60 (60.0%)	38 (67.9%)	22 (50.0%)	*χ*^2^ = 3.274	0.070
Osteoporosis	40 (40.0%)	18 (32.1%)	22 (50.0%)		
**Osteoporosis duration (years)**					
Median (min.–max.)	4 (2–7)	4 (2–6)	4 (2–7)	*U* = 1134.0	0.483
**Compression fracture**	21 (21.0%)	12 (21.4%)	9 (20.5%)	*χ*^2^ = 0.014	0.906
**Treatment received**					
**Denosumab**	56 (56.0%)	29 (51.79%)	27 (61.36%)	*χ*^2^ = 0.92	0.34
**Bisphosphonate**	26 (26.0%)	16 (28.57%)	10 (22.72%)	*χ*^2^ = 1.59	0.21
**Teriparatide**	18 (18.0%)	11 (19.64%)	7 (15.92%)	*χ*^2^ = 0.232	0.63
**Ca/vitamin D**	98 (98.0%)	56 (100.0%)	42 (95.5%)	*χ*^2^ = 2.597	^FE^*p* = 0.191
**Corticosteroid use**	43 (43.0%)	24 (51.8%)	19 (31.8%)	*χ*^2^ = 0.001	0. 97
**Duration of corticosteroid (years)**					
Median (min–max)	0 (0–6)	2 (0–6)	0 (0–6)	*U* = 975.5	0. 075
**Hospitalization**					
** Hospital admission**	40 (40.0%)	40 (71.4%)	–	–	–
** Days of admission** Mean ± SD	**(*n* = 40)**	**(*n* = 40)**	**(*n* = 0)**		
	8.83 ± 4.13	8.83 ± 4.13	– –	–	–

*SD* standard deviation, *U* Mann–Whitney test, *t* Student *t*-test, *χ*^*2*^ chi square test, *BMI* body mass index, *HTN* hypertension, *DM* diabetes mellitus, *Ca* calcium, *COVID* coronavirus disease, *p*,*p* value for comparing between COVID and non-COVID

^*^Statistically significant at *p* ≤ 0.05

Twenty-nine patients out of 56 patients who were on denosumab, 16 patients out of 26 patients on bisphosphonate, 11 patients out of 18 patients on teriparatide got COVID-19 infection.

There were no statistically significant differences between the two groups regarding serum levels of total calcium, vitamin D, phosphorus, HbA1C, creatinine, urea, and CRP as shown in Table 2.

**Table 2:** Laboratory data of the patients (*n* = 100)

	**Total (*n* = 100)**	**COVID (*n* = 56)**	**Non-COVID (*n* = 44)**	**Test of sig**	***p***
**Ferritin (µg/L)** Median (min.–max.)	**(*n* = 56)**	**(*n* = 56)**	–		
	486 (154–1213)	487 (154–1213)			
**CRP (mg/dl)** Mean ± SD	6.44 ± 3.79	6.45 ± 2.91	5.98 ± 3.02	*t* = 0.788	0.43
**HbA1C %** Mean ± SD	6.99 ± 1.80	7.01 ± 1.81	6.97 ± 1.82	*t* = 0.090	0.929
**Creatinine (µmol/L)** Mean ± SD	72.96 ± 49.67	81.0 ± 48.98	68.90 ± 40.23	*t* = 1.325	0.188
**Urea (mmol/L)** Median (min.–max.)	4.2 (2.1–42)	4.0 (2.4–38.1)	4.35 (2.1–42)	*U* = 1130.5	0.481
**Vitamin D level (nmol/L)** Median (min.–max.)	47.3(10.42–162.3)	44.7 (20–162.3)	55.0 (10.4–123.1)	*U* = 999.0	0.106
**Calcium (mmol/L)** Mean ± SD	2.32 ± 0.13	2.29 ± 0.13	2.34 ± 0.11	*t* = 1.909	0.059
**Phosphorus (mmol/L)** Mean ± SD	1.10 ± 0.24	1.08 ± 0.27	1.13 ± 0.20	*t* = 1.107	0.271

*SD* standard deviation, *t* Student *t*-test, *U* Mann–Whitney test, *χ*^*2*^ chi-square test, *CRP* C-reactive protein, *HbA1C* glycosylated hemoglobin, *COVID* coronavirus disease, *p p* value for comparing between COVID and non-COVID

^*^Statistically significant at *p* ≤ 0.05

There was a significant decrease in BMD of lumbar region and femur at 9 months as compared to baseline in the osteoporotic patients who got COVID infection, while there was a significant increase in the lumbar BMD of osteoporotic patients who did not get COVID infection after 21 months. Table 3 shows the comparison between the three studied periods according to lumbar and femur BMD.

**Table 3:** Comparison between the three studied periods according to lumbar and femur BMD

	**Baseline**	**After 9 months**	**After 21 months**	**Test of sig**	***p***
**Lumbar BMD**					
**COVID (*n* = 56)**					
Mean ± SD	0.96 ± 0.11	0.91^#^ ± 0.11	0.96 ± 0.11	*F* = 25.202^*^	< 0.001^*^
**Non-COVID (*n* = 44)**					
Mean ± SD	0.92 ± 0.11	0.92 ± 0.10	0.96^#^ ± 0.11	*F* = 10.384^*^	< 0.001^*^
**Femur BMD**					
**COVID (*n* = 56)**					
Mean ± SD	0.88 ± 0.12	0.84^#^ ± 0.11	0.89 ± 0.13	*F* = 7.677^*^	0.002^*^
**Non-COVID (*n* = 44)**					
Mean ± SD	0.89 ± 0.11	0.88 ± 0.13	0.89 ± 0.13	*F* = 1.335	0.268

*P* value for comparing between the three studied periods

*SD* standard deviation, *F F* test (ANOVA) with repeated measures (sig., bet. periods was done using post hoc test (adjusted Bonferroni)), *BMD* bone mineral density; *COVID* coronavirus disease

^#^Significant with baseline

^*^Statistically significant at *p* ≤ 0.05

As shown in Table 4, a significant bone loss (*p* < 0.001) was found between baseline and 9 months follow-up at the femoral neck and lumbar spine in group 1 (osteoporotic and osteopenic patients who got COVID infection) when compared to group 2 (osteoporotic and osteopenic patients who did not get COVID infection), with significant increase in BMD in lumbar spine after 21 months in group 2 as represented by the percentage of change in BMD.

**Table 4:** Percentage of change in bone mineral density (BMD) at femur and lumbar spine after 9 and 21 months follow-up in both groups

% change from baseline	COVID (*n* = 56)	Non-COVID (*n* = 44)	*U*	*p*
**Lumbar BMD**				
**After 9 months**				
Median (min.–max.)	− 4.68(− 19.19–19.23)	0.0 (− 16.67–12.12)	657.0^*^	< 0.001^*^
**After 21 months**				
Median (min.–max.)	0.99 (− 13.73–11.11)	5.72 (− 13.73–18.27)	727.5^*^	< 0.001^*^
**Femur BMD**				
**After 9 months**				
Median (min.–max.)	− 3.6 (− 27.3–13.1)	0 (− 17.4–12.9)	836.5^*^	0.006^*^
**After 21 months**				
Median (min.–max.)	0 (− 14.55–46.38)	2.39 (− 16–8.33)	1146.5	0.552

*P* value for comparing between COVID and non-COVID

*U* Mann–Whitney test, *BMD* bone mineral density, *COVID* coronavirus disease

^*^Statistically significant at *p* ≤ 0.05

There was a significant difference between the three subgroups of COVID patients regarding percentage of change in BMD in the femur and lumbar spine after 9 months with the most decrease in the severe subgroup (Table 5).

**Table 5:** Comparison between the three subgroups (according to severity) of COVID infection regarding percentage of change in bone mineral density (BMD) at femur and lumbar spine after 9 months

	**Mild**	**Moderate**	**Severe**	**Test of sig**	***p***
**Lumbar BMD**					
**COVID (*n* = 56)**					
Median	− 2.09 (− 5.59 to 19.46)	− 6.11 (− 10.98 to 7.38)	− 15.14 (− 19.25 to − 9.21)	*H* = 37.69*	< 0.00001*
**Femur BMD**					
**COVID (*n* = 56)**					
Median	− 1.02 (− 9.46 to 13.46)	− 3.84(− 9.41 to 1.71)	− 7.78(− 27.09 to − 1.95)	*H* = 16.79*	0. 00,023*

*p* value for comparing between comparing between the three subgroups of COVID (according to severity)

*H* Kruskal–Wallis test, *BMD* bone mineral density, *COVID* coronavirus disease

^*^Statistically significant at *p* ≤ 0.05

When we compared percentage of change in BMD in the femur and lumbar spine after 9 months between COVID patients who received corticosteroids and non-COVID patients who were treated by corticosteroids for other comorbidities, e.g., rheumatoid arthritis and systemic lupus erythematosus, we found that there was a more significant lowering of BMD in COVID group than non-COVID in both the femur and lumbar spine (*p* = 0.008 and *p* = 0.002 respectively).

## Discussion

In our retrospective study, the decrease in BMD was significantly higher after 9 months in patients who got COVID-19 infection than in patients who did not get COVID-19 infection which reflects the burden of this viral disease on bone homeostasis that could be attributed to either the inflammatory nature of the disease and/or the side effects of treatment modalities for this acute sometimes serious infection. Increased COVID-19 severity is associated with a greater decrease in BMD.

Hospitalized COVID-19 patients require specialized care because of numerous risk factors, which include glucocorticoid medication, various comorbidities, and high levels of inflammatory cytokines [5, 6].

COVID-19 infection is associated with high inflammatory cytokines and prolonged immobilization especially in severely ill patients, in addition to corticosteroid treatment which may lead to increase bone loss and bone resorption [3].

An in vitro study revealed that the severe acute respiratory syndrome coronavirus (SARS-CoV) protein 3a/X1 accelerates osteoclast differentiation from monocyte/macrophage progenitors, increases the production of RANKL and inflammatory cytokines including tumor necrosis factor alpha (TNF-α), and promotes osteoclastogenesis by direct and indirect mechanisms [7].

The osteo-metabolic phenotype of COVID-19 is characterized by acute hypocalcemia and chronic hypovitaminosis D and high prevalence of morphometric vertebral fractures [8–10].

Berktaş et al. assessed the BMD of hospitalized COVID-19 patients at diagnosis and at follow-up visits; BMD was retrospectively measured by quantitative CT. They found that secondary osteoporosis may occur as a post-acute sequelae of COVID-19 [11].

Nurkovic et al. concluded that in Novi Pazar city, people with COVID-19 infection had increased risk of osteoporosis [12].

Qiao et al. studied the effects of severe SARS-CoV-2 infection on bone metabolism in an established golden Syrian hamster model for COVID-19; they found that the bone loss is associated with SARS-CoV-2-induced cytokine dysregulation, as the circulating pro-inflammatory cytokines not only upregulate osteoclastic differentiation in bone tissues but also trigger an amplified pro-inflammatory cascade in the skeletal tissues to augment their pro-osteoclastogenesis effect [13].

Since COVID-19 impairs bone health patients with several risk factors for bone loss, patients who are hospitalized for COVID-19 should be monitored, and preventive treatment may be necessary. Age over 50, decreased mobility, malnutrition, hypocalcemia, elevated serum pro-inflammatory cytokines, and usage of corticosteroids are some of these risk factors [14, 15].

Osteoclastogenesis caused by the SARS-CoV 1 virus has been proven in vitro. There have also been reports of suppressed osteogenic differentiation and reduced fracture healing as a result of miR-4485 being overexpressed as a result of SARS-CoV-2 [16, 17].

It was reported that postmenopausal women under pharmacologic treatment for osteoporosis do not seem to be at high risk of symptomatic/severe COVID-19 and denosumab did not appear to be a risk factor for COVID-19 [18].

This study has several limitations due to its retrospective design, relatively small number of cases, and also the short time of follow-up.

## Conclusion

COVID-19 may have deleterious effect on BMD in osteoporotic patients. It is recommended to assess BMD in osteoporotic/osteopenic patients who got COVID infection to detect if there is an increased risk of fracture which may necessitate post-COVID change in the therapeutic intervention plan.

## Supplementary Information

**Additional file  1.** STROBE Statement—checklist of items that should be included in reports of observational studies.

## Data Availability

The data will be available upon reasonable request.

## Abbreviations

BMD: Bone mineral density
CDC: Center for Disease Control and Prevention
COVID-19: Coronavirus disease 2019
DXA: Dual energy x-ray absorptiometry
HbA1c: Glycosylated hemoglobin
ICH GCP: International Council for Harmonisation, Good Clinical Practice
PaO_2_/FiO_2_: Ratio of arterial partial pressure of oxygen to fraction of inspired oxygen
RANKL: Receptor activator of NF-kB ligand
SARS-CoV: Severe acute respiratory syndrome coronavirus
SpO_2_: Oxygen saturation
TNF-α: Tumor necrosis factor alpha
